# Supplementary material for: Complex regulation of Cav2.2 N-type Ca2+ channels by Ca2+ and G-proteins
Source: PLoS One. 2025 Feb 7;20(2):e0314839. doi: 10.1371/journal.pone.0314839 (PMC11805433; doi:10.1371/journal.pone.0314839)
Supplement: Attachment — Submitted filename: Rebuttal.docx [file pone.0314839.s002.docx]

We thank the reviewers for their careful review of our manuscript. We have addressed their concerns as outlined below:

Reviewer #1: 
1. In Figure 3, why is the voltage-dependent facilitation (VDF) of ICa so strong, similar to that of IBa, while it appears much weaker than IBa in Figures 1 and 2? The differences should be clearly demonstrated.

In Figs.1,2,4 the voltage protocol involves analyzing the effect of a +60 mV prepulse on currents evoked at different test voltages (P1, P2). Here, I_norm_ represents each test current normalized to the maximal current evoked by the second (P2) test pulse. In Fig.3,5,6 we analyze the effect of varying prepulses on test currents evoked by a single test voltage, before (P1) and after (P2) the prepulse. Here I_norm_ represents the P2 tail current divided by the P1 tail current. Therefore, the two protocols provide two different measures of VDF. To clarify the differences in these protocols, we modified the corresponding figure legends with these details.

2. To gain a deeper understanding of the molecular mechanism, it would be beneficial to compare the results of VDF experiments in channels with membrane-tethered β2a or cytosolic CaV β subunits, such as β1a or β3. I recommend testing the key findings in channels with cytosolic β subunits.

The focus of our study was on uncovering the influence of Ca^2+^ permeation on G-protein modulation of Cav2.2. Based on previous work (PMID: 36374183), we would expect that the difference in VDF of I_Ca_ and I_Ba_ would indeed depend strongly on the identity of the CaV β subunit. We agree that comparing results obtained with β1a or β3 with those we obtained with β2a would be interesting but feel that such experiments are beyond the scope of our study. Also in response to reviewer 2, we have re-written the last paragraph of the discussion which adds reference to comparative studies of Ca_v_β subunits as an important next step:

“PIP2 has complex actions on Ca_v_2 channels, causing both a stimulation and inhibition of function [41]. According to one model, PIP2 binds to an “R” domain which, like G-proteins, causes channels to enter a “reluctant” (i.e., inhibited) mode of gating at intermediate voltages. In contrast, PIP2 binding to a stimulatory “S” domain is required for channel activation [41]. Structural and functional studies show that the S domain likely corresponds to a PIP2 binding site in domain II S4 [51-53]. Additionally, a second site in the cytoplasmic I-II linker is important for stimulatory effects of PIP2 on Ca_v_2.2 channels containing the cytosolic β_2c_ but not the membrane-tethered β_2a_ subunit [53]. Apparently, the palmitoylation of β_2a_ allows it to compete with PIP2 binding to the “S” domain, perhaps biasing its interaction with the “R” domain [53-55]. Compared to Ca_v_2.2 channels with other β subunits, those containing β_2a_ are less sensitive to the stimulatory effects, and more sensitive to the inhibitory effects, of PIP2; an increase in current density is seen upon Gα_q_-linked receptor activation of β_2a_-containing channels [54, 55]. The conversion of “reluctant” channels to “willing” channels upon Dr-VSP activation could contribute to the reduced VDF of *I_Ca_* if “willing” channels represent a “pre-facilitated” state. Based on this logic, the effects of Gα_q_-mediated PIP2 depletion on VDF are expected to differ for Ca_v_2.2 channels containing β_2a_ vs. cytosolic β subunits (i.e., β_2c_ or β_3_), which may further diversify the modulatory properties of these channels in neurons. PIP2 has been shown to support VDF of Ca_v_2.2 channels in hypothalamic neurons [56]. Moreover, Ca^2+^ and PIP2 have been found to strengthen Gβγ-mediated inhibition of Ca_v_2 channels [57-59]. Future studies are needed to dissect the mechanisms whereby alterations in PIP2 enable gating transitions that underlie VDF, and the interplay of Ca^2+^, G-proteins, and Ca_v_β subunits in this process.”

3. Please correct "β2A" to "β2a" in the first paragraph of the Materials and Methods and Results sections. Done

4. Kindly check the spacing between words, numbers, and units throughout the text. Done.

Reviewer #2

The manuscript could contribute substantially to the calcium channel field if critical context in data interpretation on several issues is added by expanding discussion of several previous studies.

Context Issues:

1) CaV2 model of 2 PIP2 binding sites: The authors do not address previous findings that CaV2 channels appear to have 2 PIP2 binding sites that exert different effects on CaV2.2 activity. It may not appear obvious that their data may arise from both or just one PIP2 binding site. Previously, Wu et al (2002) presented a strong argument for two PIP2 binding sites on CaV2 channels: one site regulates whether channels open with “willing” voltage sensitivity (S site) and the second, lower affinity PIP2 binding site (R) promotes reluctant gating that is voltage-dependent where strong voltage pulses facilitate current amplitude to “willing” kinetics and amplitude. Loss of PIP2 from the R site enhances current amplitude promoting willing gating such that a prepulse no longer facilitates current amplitude. Thus, conditions that shift equilibrium in favor of PIP2 bound at the R site would be predicted to promote VDF following a prepulse. Loss of PIP2 at this site shifts the activation to more negative voltages and the channels undergo rapid activation and no VDF. Thomas et al’s should discuss their data in context of the Wu et al model since their data are complimentary to this model.

2) Palmitoylated β2a: The Hille, Rittenhouse, & Suh labs have each published findings that when β2a is expressed in HEK cells, its 2 palmitoyl groups, located on vicinal cysteines, appear occupy the “S” PIP2 binding site and confer resistance to current inhibition by M1R/Gq signaling and PIP2 breakdown. Under Thomas et al’s recording conditions, as with the above studies, the palmitoylated β2a will block the PIP2 “S” putative binding site leaving modulation of the voltage-dependent “R” site. The authors need to point this out since it is directly relevant to their data interpretation as mentioned above.

Thank you for suggesting this line of discussion. In response to points 1 and 2, we modified the last paragraph of the discussion (see response to Reviewer 1, point 2).

3) Previous expts with BAPTA & Ca2+: BAPTA can interact with PIP2 protecting it from breakdown. The authors need to look at Shapiro et al 1994 in Neuron, Rousset et al 2004a (JBC) and 2004b (FEBS Letters). Each of these studies address biophysical aspects of interactions among Ca2+, BAPTA, G-proteins. The data in these papers may help further mechanistic insight in the differences in VDF with Ba2+ vs Ca2+.

We did not observe any effects of BAPTA (Fig.6B,D), which suggested that BAPTA was not acting to modify PIP2 levels relevant for VDF of I_Ca_. Nevertheless, we appreciate the suggestion and incorporated citations of these references in the new last paragraph of the discussion.

4) Alternative theories: while the authors conclude that PIP2 regulates ICa2+, rather than CaM kinases or G-protein mediated signaling cascades, the authors should not rule out a role for enzymes directly activated by Ca2+ and/or PIP2 without Gβγ activity. Good to cover all possibilities.

This is an excellent point, and one that we already mentioned in the Discussion, paragraph 2: “Considering that Ca^2+^ can increase the enzymatic activity of PLC [45, 46], Ca^2+^ influx through Ca_v_2.2 could amplify the effects of Gα_q_ coupling to PLC, leading to greater reductions in PIP2 levels and reduced channel function than when using Ba^2+^ as the permeant ion.”

Major Issues
The prepulse voltage used varies with different figures. For example, Figs 1 &2 use a prepulse to +60 mV whereas Fig 7 it is +20 mV. The I-FF figs were apparently gathered from different prepulse voltages, e.g., Figs. 3F,5D, 6D plot I-FF80, whereas Figs 1G,2G & 4G, plot I-FF60, and Fig 7 data are plotted P2/P1 vs P2-P1/P1as with the other figures. It is not clear why in Fig. 7 the Pre pulse is only to +20 mV. The authors need to explain the thinking behind the different voltages and calculations. Explain why they used different voltages since it adds an additional variable when comparing experimental data.

We regret if this was unclear, but we explained the use of the different prepulse voltage in Fig.7 in the last paragraph of the Results section: “To enable VSP activation, we modified our voltage protocol to include a +120-mV VSP-activating pulse prior to the P1 test pulse and VDF was measured as the ratio of the P2/P1 pulses with an intervening +20 mV prepulse (Fig.7A). A more modest depolarizing prepulse was used in these experiments to avoid additional activation of the VSP.”

As described in our response to Reviewer 1, point 1, Fig.3,5,6 and Fig.1,2,4 utilize distinct voltage protocols to measure different aspects of VDF (pre-pulse dependence vs test-pulse dependence). As such, we believe the different metrics for VDF (FF80 vs FF60) are warranted, especially since we do not compare data obtained with different voltage protocols via statistical analysis.

For Fig. 7 include 2 graphs like those in Figs. 1C & F. Done.

Activation kinetics of current traces were not measured despite it being a hallmark of G-protein modulation. The authors have the traces. The activation kinetics, e.g. time to peak or τo measurements should support changes in current amplitudes following prepulses. The extra data would strengthen their observation that Ca2+ vs Ba2+ current modulation show different characteristics.

We respectfully chose not to perform this analysis since we expect the differences in kinetics of I_Ca_ and I_Ba_ to be rather subtle since both undergo kinetic slowing due to Gβγ- mediated inhibition, with faster onset following the depolarizing prepulse. Note the similar kinetics of I_Ca_ and I_Ba_ in Figs. 1,2. Moreover, the focus of our study was not on Gβγ- mediated inhibition, but rather how Gβγ- independent VDF is altered in Ca2+ vs Ba2+.

Minor Issues
1. Everywhere Gi is written should be changed to Go/i. Indeed, Go may mediate more VDF than Gi. Done.

2. State where the amplitude of current traces were measured, e.g., at the end of the test pulse, the peak tail current?

We clarified in the legends to Figs.1,2,4 that the current amplitudes were measured at the end of the 10-ms test pulse and that in Figs.3,5-7 that the tail current amplitudes were measured.

3. Explain why data are expressed as median rather than the mean.

The median was reported in cases where the data were non-normally distributed whereas the mean was reported when data were normally distributed. We added clarification to the legends for the statistically analyzed data as to whether the bar in graphs of grouped data represents the median or mean.

4. The figures need more detail: what is the test pulse for all figs where current amplitudes and FF were calculated, e.g, for Fig 2 the test voltage was 0 mV but it is not clear for Fig. 2 C & F.

We have indicated that the test voltage was 0 mV in the corresponding figure legends.

5. In Fig. 1, the IBa individual P1 traces are so small that the currents can’t be compared directly to the P2 traces. If possible, replace the recording for one where the current amplitudes are greater.

Done.

6. The I-FF data would be clearer if in Figs 1G, 2G, #F & 4G graphs use symbols other than circles, e.g. squares or triangles to distinguish the difference in data graphed compared to C & F.

Done.

Additional changes:

--Added complete datasets from which figures were generated as Supporting Information

--Added Acknowledgements section

-- Added these references:

54.	Suh BC, Kim DI, Falkenburger BH, Hille B. Membrane-localized beta-subunits alter the PIP2 regulation of high-voltage activated Ca2+ channels. Proc Natl Acad Sci U S A. 2012;109(8):3161-6. Epub 20120202. doi: 10.1073/pnas.1121434109.

55.	Heneghan JF, Mitra-Ganguli T, Stanish LF, Liu L, Zhao R, Rittenhouse AR. The Ca2+ channel beta subunit determines whether stimulation of Gq-coupled receptors enhances or inhibits N current. J Gen Physiol. 2009;134(5):369-84. doi: 10.1085/jgp.200910203.

57.	Shapiro MS, Wollmuth LP, Hille B. Angiotensin II inhibits calcium and M current channels in rat sympathetic neurons via G proteins. Neuron. 1994;12(6):1319-29. doi: 10.1016/0896-6273(94)90447-2.

58.	Rousset M, Cens T, Gouin-Charnet A, Scamps F, Charnet P. Ca2+ and phosphatidylinositol 4,5-bisphosphate stabilize a Gbeta gamma-sensitive state of Ca V2 Ca 2+ channels. J Biol Chem. 2004;279(15):14619-30. Epub 20040113. doi: 10.1074/jbc.M313284200.

59.	Rousset M, Cens T, Vanmau N, Charnet P. Ca2+-dependent interaction of BAPTA with phospholipids. FEBS Lett. 2004;576(1-2):41-5. doi: 10.1016/j.febslet.2004.08.058.
